# Preparation of Mangosteen Peel Extract Microcapsules by Fluidized Bed Spray-Drying for Tableting: Improving the Solubility and Antioxidant Stability

**DOI:** 10.3390/antiox11071331

**Published:** 2022-07-06

**Authors:** Sriwidodo Sriwidodo, Reza Pratama, Abd. Kakhar Umar, Anis Yohana Chaerunisa, Afifah Tri Ambarwati, Nasrul Wathoni

**Affiliations:** 1 Department of Pharmaceutics and Pharmaceutical Technology, Faculty of Pharmacy, Universitas Padjadjaran, Sumedang 45363, Indonesia; abd17002@mail.unpad.ac.id (A.K.U.); anis.yohana.chaerunisaa@unpad.ac.id (A.Y.C.); afifah17007@mail.unpd.ac.id (A.T.A.); nasrul@unpad.ac.id (N.W.); 2 Department of Pharmaceutics and Pharmaceutical Technology, Faculty of Pharmacy, Universitas Bhakti Kencana, Bandung 40614, Indonesia; reza.pratama@bku.ac.id

**Keywords:** antioxidant stability, mangosteen peel extract, extract microencapsulation, α-mangosteen

## Abstract

Mangosteen fruit has been widely consumed and used as a source of antioxidants, either in the form of fresh fruit or processed products. However, mangosteen peel only becomes industrial waste due to its bitter taste, low content solubility, and poor stability. Therefore, this study aimed to design mangosteen peel extract microcapsules (MPEMs) and tablets to overcome the challenges. The fluidized bed spray-drying method was used to develop MPEM, with hydroxypropyl methylcellulose (HPMC) as the core mixture and polyvinyl alcohol (PVA) as the coating agent. The obtained MPEM was spherical with a hollow surface and had a size of 411.2 µm. The flow rate and compressibility of MPEM increased significantly after granulation. A formula containing 5% *w/w* polyvinyl pyrrolidone K30 (PVP K30) as a binder had the best tablet characteristics, with a hardness of 87.8 ± 1.398 N, friability of 0.94%, and disintegration time of 25.75 ± 0.676 min. Microencapsulation of mangosteen peel extract maintains the stability of its compound (total phenolic and α-mangosteen) and its antioxidant activity (IC50) during the manufacturing process and a month of storage at IVB zone conditions. According to the findings, the microencapsulation is an effective technique for improving the solubility and antioxidant stability of mangosteen peel extract during manufacture and storage.

## 1. Introduction

Indonesia is one of the world’s leading producers of mangosteen fruit (*Garcinia mangostana* L.), both fresh and processed into canned products and juices [1,2]. During mangosteen processing, the peel is separated and discarded as industrial waste, even though it offers numerous benefits. Mangosteen peel can be used as a food, supplement, cosmetic, and medicine because it contains many therapeutic compounds and antioxidants [3,4,5,6,7,8,9]. Mangosteen peel is rarely ingested raw due to its astringent taste. The major antioxidant components in mangosteen peel, xanthones, have poor water solubility, and direct usage reduces their efficiency since they can be degraded or oxidized during processing and storage. According to reports, the microencapsulation technique can overcome these challenges [10,11,12].

Microencapsulation is the process of encapsulating tiny solid, liquid, or gaseous particles in a polymeric substance. The polymer material can modulate odor, taste, volatility, and reactivity, as well as enhance the extract material’s stability and solubility [13,14,15,16]. Many factors affect the quality of microcapsules, including the preparation technique and the type of coating material [17]. Fluidized bed microencapsulation is the prevalent approach and offers several advantages, including low cost, minimal dust pollution, high uniformity, high encapsulation efficiency, and excellent stability [18,19]. The polymer utilized further enhances the quality and stability of the microencapsulation; hence, choosing the proper base polymer should be performed [17,20].

Several natural polymers have been used as core and coating materials in microencapsulation. One of them is HPMC, which is known to be biodegradable, biocompatible, renewable, and low in toxicity [21,22]. HPMC has been used as a mixture with active ingredients to increase solubility and extend drug contact time due to its hydrophilic and mucoadhesive properties [23,24,25]. This cellulose derivate has also demonstrated substantial potential to alleviate GI irritation caused by APIs known to induce diarrhea, ulcers, nausea, or vomiting [23]. Since HPMC is hygroscopic, particularly after drying, it is typically mixed with other polymers [26,27] or coated [23]. As a coating material, PVA offers several advantages, including biodegradability, biocompatibility, and good mechanical properties [28]. The PVA film is both water- and heat-resistant, which contributes to the core material’s stability [29]. The study by Wu et al. reveals that employing PVA as a coating can minimize the size of the microencapsulation while also providing protection against coalescence [30]. The encapsulation efficiency of PVA is also reported to be high [31]. As a result, we employed HPMC as the core material’s binder and PVP as the microspheres’ coating. In this work, we investigated the stability and antioxidant activity of mangosteen peel extract during its transformation into microcapsules and, lastly, tablets. The characteristics of microcapsules, granules, and tablets were also studied.

## 2. Materials and Methods

### 2.1. Materials

We used the dry mangosteen peel powder obtained from Subang, Indonesia, ethanol 96% (PT. Brataco, Indonesia), polyvinyl alcohol (PVA) (Sigma Aldrich, St. Louis, MO, USA), polyvinylpyrrolidone (PVP K30) (Sigma Aldrich, St. Louis, MO, USA), citric acid (PT. Brataco, Indonesia), sodium citrate (PT. Brataco, Indonesia), xanthan gum (Sigma Aldrich, St. Louis, MO, USA), sucrose (PT. Brataco, Indonesia), carboxymethylcellulose sodium (Sigma Aldrich, St. Louis, MO, USA), hydroxypropylmethylcellulose (HPMC) (Sigma Aldrich, St. Louis, MO, USA), 1,1-diphenyl-2-picryl-hydrazyl (DPPH) (Sigma Aldrich, St. Louis, MO, USA), Mg stearate (Sigma Aldrich, St. Louis, MO, USA), Amprotab (Sigma Aldrich, St. Louis, MO, USA), talcum (Sigma Aldrich, St. Louis, MO, USA), and lactose (PT. Brataco, Indonesia). 

### 2.2. Mangosteen Peel Extraction

The 25 kg of fine dry mangosteen peel powder was placed in the macerator (Extraction and Concentration Machine TD-300, China) and soaked in aqueous ethanol solution 70% (*v*/*v*) until all the powder was wetted, then ethanol was added again until the powder was completely submerged. This procedure was carried out for 4 days, with a replacement of solvent every 24 h. Each macerate was filtered and mixed before being evaporated to make a thick extract in a rotary evaporator (BUCHI Rotavapor R-300, PT. BUCHI, Indonesia) at 40 °C and 30 rpm. 

### 2.3. Phytochemical Screening, Standardization, Thin-Layer Chromatography Profile, and Antioxidant Activity of Mangosteen Peel Extract 

Phytochemical screening was conducted to determine the secondary metabolite content of mangosteen peel extract (MPE). The phytochemical screening included alkaloids, tannins, polyphenols, flavonoids, quinones, saponins, monoterpenes, sesquiterpenes, steroids, and triterpenoids. Standardization was also carried out to determine the quality of MPE. Types of inspection performed were organoleptic, ethanol-soluble extract content, water-soluble extract content, total ash content, acid-insoluble ash content, and drying shrinkage.

The thin-layer chromatography profile of MPE was examined using a silica plate GF 254 with a mixed mobile phase of chloroform and ethyl acetate (9:1). After the chamber was saturated, 5% *v*/*v* MPE solution in methanol was applied to the starting line on the silica plate and then waited for the mobile phase to reach the finish line on the silica plate. The retention factor value of each spot was recorded.

The antioxidant activity of MPE was determined using the DPPH reagent. The sample and positive control (ascorbic acid) were prepared to as much as 100 µL with a concentration range of 10–50 and 1–5 ppm, respectively. A total of 0.1 mL of DPPH reagent (0.2 mg/mL in ethanol) was added to the sample and the positive control. The sample and positive control were allowed to stand for 30 min in the dark (25 °C), after which the absorbance was measured at a wavelength of 517 nm (Epoch™ Microplate Spectrophotometer, BioTek Instrument, Inc., Winooski, VT, USA). The IC50 value was calculated from the linear equation of %inhibition vs. concentrations, where %inhibition was obtained using the following equation:

(1) $$\%\;\mathrm{inhibition}=\frac{A_{Control}-A_{Sample}}{A_{Control}}\times 100\%$$

### 2.4. Total Phenolic and α-Mangosteen Content of Mangosteen Peel Extract

Total phenolic of MPE was determined using gallic acid as the standard. The standard was made into several variations of concentration (200–600 ppm). The extract solution was prepared by dissolving 10 mg of MPE in 10 mL of methanol. One mL of the extract solution and each concentration of the standard solution was put into a dilution tube, then 5 mL of Folin–Ciocalteau reagent was added. The preparations were mixed for 8 min, then 4 mL of 1% *v*/*v* NaOH was added. The absorbance of the extract solution and the standard were measured at a wavelength of 256 nm (Epoch™ Microplate Spectrophotometer, BioTek Instrument, Inc., Winooski, VT, USA).

Determination of the α-mangosteen content was carried out using high-performance liquid chromatography (HPLC) (Waters Alliance HPLC, Waters Corporation, USA). The standard solution of α-mangosteen was made into several concentrations in the range of 10–50 ppm to create the standard curve. The extract solution (10 mg/mL in methanol) was filtered using a syringe filter and then inserted into the HPLC sample tube. The column used was C18. The mobile phase used was methanol and aquades (95:5 *v*/*v*). The injection volume was set to 10 L with a flow rate of 1 mL/min. The retention time of α-mangosteen was 10 min and was measured at a wavelength of 318 nm using a UV detector.

### 2.5. Mangosteen Peel Extract Microencapsulation

Microcapsules of MPE (MPEM) were prepared using a Fluid Bed Spray Dryer (PMS FBD5, Armitec, Thailand). Outlet temperature was set at 37.2 °C, with a spray interval of 10 s, product temperature of 37.8 °C, inlet temperature of 37.8 °C, and spray rate of 20.5 rpm. The core material (microspheres) was prepared by spraying a mixture of 20% *w*/*v* HPMC and 33.3% *v*/*v* MPE (600 mL) onto 1.5 kg of lactose. After the mixture was dry and homogeneous, the microspheres were again sprayed with 15% *w*/*v* polyvinyl alcohol in 450 mL of water as a coating agent. The thin-layer chromatography profile and IC50 of MPEM were then determined using the previously described method.

### 2.6. Characterization of Mangosteen Peel Extract Microcapsule

#### 2.6.1. Moisture Content 

A total of 1 g of MPEM was placed in a dish on a moisture balance device (Moisture Analyzer MA 50.R, Radwag, Miami, FL, USA). The temperature was set at 105 °C. Drying loss was recorded after the tool showed constant weight during heating. 

#### 2.6.2. Flow Rate and Angle of Repose

A total of 25 g of MPEM was placed in the funnel of the flowmeter (GTB Series, Erweka, Langen, Germany). The MPEM flow rate was determined by observing the time it takes for MPEM to pass through the funnel until it runs out. The angle of repose was obtained by measuring the diameter and height of the MPEM pile formed. 

#### 2.6.3. Compressibility Index

A total of 25 g of MPEM was put into a measuring cup contained in the volumenometer (Tapped Density Tester, Erweka SVM 221, Erweka). The compressibility index of MPEM was determined by the final volume of the microcapsule after 500 beats.

#### 2.6.4. Shape, Morphology, and Particle Size

The shape and surface morphology of MPEM were observed using a scanning electron microscope (SEM) (JSM-6360, Jeol, Tokyo, Japan) with 500× magnification. The particle size of MPEM was then determined using a particle size analyzer (Horiba SZ-100, Horiba Ltd., Kyoto, Japan). The test was carried out by dispersing the sample in a phosphate buffer at pH 6.8, after which a 1 mL sample was taken for testing.

#### 2.6.5. Solubility or Sedimentation Volume

A total of 5 g of MPEM was dissolved in 100 mL of distilled water and then stirred for 20 s. Sedimentation volume was measured after 15 min.

### 2.7. Tablet Formulation and Evaluation

MPEM tablets were made by mixing MPEM and internal phase materials (binder, disintegrant, and filler). The wet granulation method was used for the granulation. After the granules were formed, the external phase (lubricant, glidant, and filler residue) was added to the granule mass. The optimal binder was chosen using a formula optimization method, as indicated in Table 1. The amount of MPEM used was 435 mg, equivalent to 30 mg of ascorbic acid (based on the IC50 value). The granule mass was then molded with a diameter of 0.9 cm and a thickness of 0.33 cm. The total weight per tablet was 750 mg.

The characteristics of the granules and tablets were evaluated to determine the quality of the tablets in each test formula. The thin-layer chromatography profile and IC50 of the best formulas were determined using the previously described method. The examined granules’ properties were drying shrinkage, flow rate, angle of repose, and compressibility index using the same method for evaluating the quality of the microcapsules. The tablets evaluations were as follows.

#### 2.7.1. Organoleptic

Organoleptic examination of MPEM tablets was carried out by observing the shape, color, and aroma of the 10 whole tablets. To assess the taste, the tablet was crushed into a powder and a certain amount of the powder was then tasted. This test was assessed by 8 people of the research project team.

#### 2.7.2. Hardness Test 

A hardness test was performed by placing the tablet on the hardness tester (Monsato VMT, Vinsyst Technologies, Mumbai, India). The hardness tester applied increasing pressure periodically until the tablet cracked. The tablet hardness was then recorded in the N unit. The number of samples used in this procedure was 10 tablets.

#### 2.7.3. Size and Weight Uniformity

A total of 20 tablets were prepared to check the uniformity of the tablets’ size and weight. Tablet dimension (diameter and thickness) was measured using a caliper (Vernier Caliper, Tricle Brand, Shanghai, China), while the tablet weight was weighed using an analytical balance (Mettler Toledo ME204, PT. Mettler-Toledo, Bekasi City, Indonesia). Average dimensions and weight were then calculated along with the standard deviation.

#### 2.7.4. Friability Test 

The friability test was carried out by placing 20 weighed tablets into the friability tester (Biobase TFT-2, Biobase Group, Jinan, China). The rotational speed of the friabilator was set at 400 rpm for 15 min. After that, the tablets were removed and reweighed to obtain the average weight.

#### 2.7.5. Disintegration Test 

The disintegration test was carried out using a disintegration tester (Disintegration Tester TDT-2IM, Zhengzhou, China) with a water medium at 37 ± 2 °C. Disintegration time was recorded when the tablets in the basket were completely crushed.

### 2.8. Statistical Analysis

The paired T-test method was used to assess the significance of the change in antioxidant activity (IC50) in each intermediate product. The data were statistically analyzed using the Statistical Package for the Social Sciences (SPSS) version 22 (IBM Corporation, New York, NY, USA).

## 3. Results and Discussion

### 3.1. Phytochemical Screening and Standardization of Mangosteen Peel Extract

The yield of the extract obtained was 12.16% *w*/*w* (weight of simplicia 25 kg, extract weight 2973 kg). Eight secondary metabolites were successfully discovered in the simplicia of mangosteen peel. Following the methanol extraction method, it was found that MPE includes six secondary metabolites (see Table 2). It has been reported that MPE does contain many alkaloids, polyphenols, flavonoids, tannins, saponins, and quinones [32]. Therefore, MPE is known to have high antioxidant properties [3,33]. The extract was a thick dark liquid with a chelate odor and a bitter taste. The high tannin content is responsible for the bitter taste and chelate [34].

Based on the standardization process, it was found that the obtained MPE followed the desired specifications (see Table 3).

### 3.2. Total Phenolic and α-Mangosteen Content of Mangosteen Peel Extract

**Figure 1:**
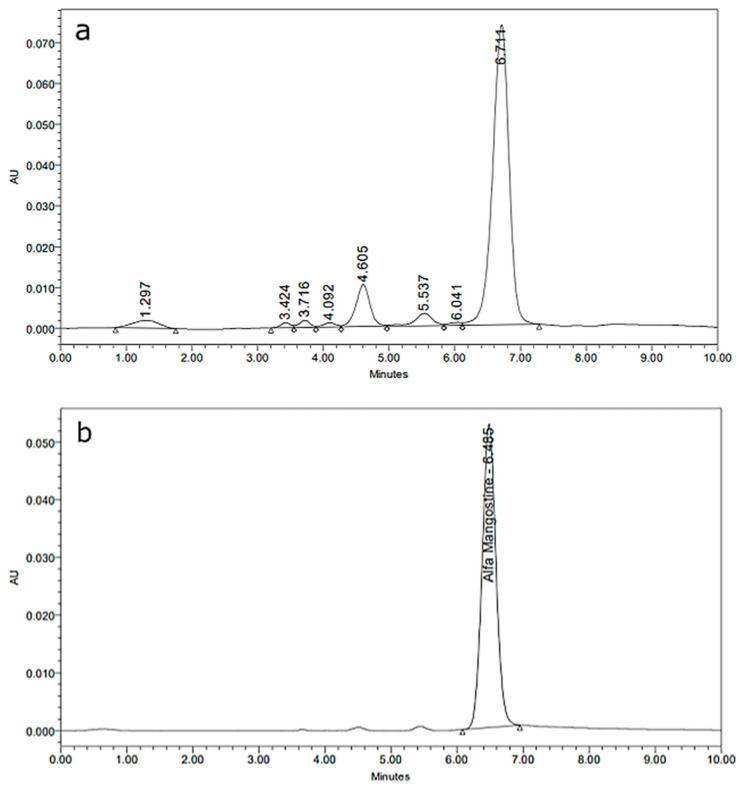
Chromatogram of mangosteen peel extract (**a**) and standard α-mangosteen (**b**).

MPE had a higher total phenolic content than that in the reported literature [34,36]. After being made into tablets, the phenolic content did not change significantly. The concentration of α-mangosteen in MPEM tablets was also high, see Table 4. This is higher than the reported data (12.06% α-mangosteen in ethyl acetate extract of mangosteen peel) [37]. Based on this percentage, the amount of α-mangosteen contained in one MPEM tablet was ~70 mg. The chromatogram of MPE and standard α-mangosteen can be seen in Figure 1.

#### 3.2.1. Physical Properties

**Figure 2:**
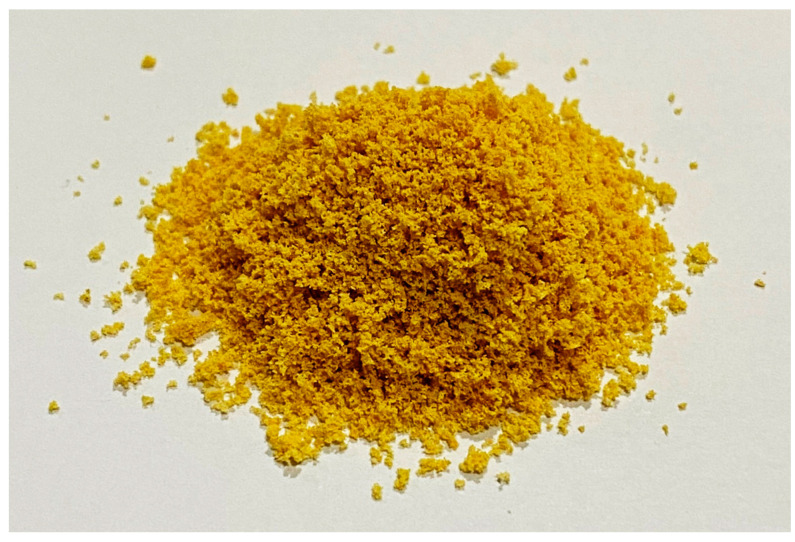
Mangosteen peel extract microcapsules.

The obtained MPEM had acceptable physical properties, see Table 5. Based on the angle of repose value, the flowability and compressibility of MPEM were fair (>30° and >15, respectively) and could be improved through granulation [38]. The MPEM also showed good solubility in water (5 g/100 mL) [39] and did not show any precipitate from 15 min to 1 day of storage. The appearance of MPEM can be seen in Figure 2. Although the PVA coating was water-resistant when dry, it did not hinder the solubility of MPEM. PVA has hydrophilic and hydrophobic groups that can act as surfactants to improve solubility [40].

#### 3.2.2. Scanning Electron Microscope (SEM) and Particle Size

**Figure 3:**
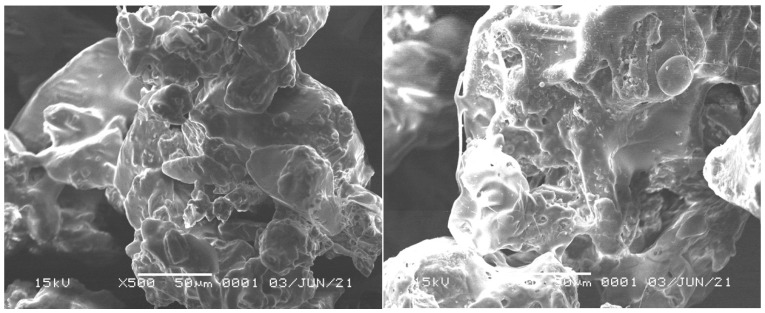
Morphological observation of mangosteen peel extract microcapsules.

The morphology of the mangosteen rind extract microcapsules was observed using a scanning electron microscope (SEM) with 500× magnification. Figure 3 shows that the particle size after coating (right) did not increase appreciably when compared to before coating (left). In both images, it can be seen that the MPEM surface was rough, hollow, and not crystalline. This situation may affect the solubility of MPEM. The particles tend to be round and connected. As reported by Ekdahl et al., the particles formed from the spray-drying process have surface characteristics that are hollow and amorphous. This might be owing to the evaporation of the solvent that was previously bonded to the particle surface and evaporated during the heating and drying process in fluidized bed spray-drying, causing voids to appear on the surface [41]. The particle size of MPEM was measured using a particle size analyzer (PSA) (Horiba SZ-100, Horiba Ltd., Kyoto, Japan) and the particle size of the mangosteen rind extract microcapsules was 411.2 µm.

### 3.3. Characterization of Granules

Drying shrinkage was used to ensure the stability of the preparation storage, and the smaller the water content, the better the quality of the granules. The smallest water content contained in MPEM granules was found in formula F3 with a LOD of 1.74%. 

The flow rate is the time required for the granules to flow through a funnel-shaped device (flowmeter). Based on the test result, the MPEM granules in formula F3 had excellent flow properties (see Table 6) because they had an angle of repose of <30° [42] and flow rate of >10 g/s [43].

The compressibility test was intended to see the decrease in granule volume due to tapping accompanied by vibration. MPEM granules in the F3 formula had a good compressibility index with a value below 15 [44]. Based on the four test parameters, the formula with the best granule properties was a formula containing 5% *w*/*w* PVP K30 as a binder. This granulation process has also been shown to increase the flowability and compressibility of MPEM.

### 3.4. Tablet Evaluations

**Figure 4:**
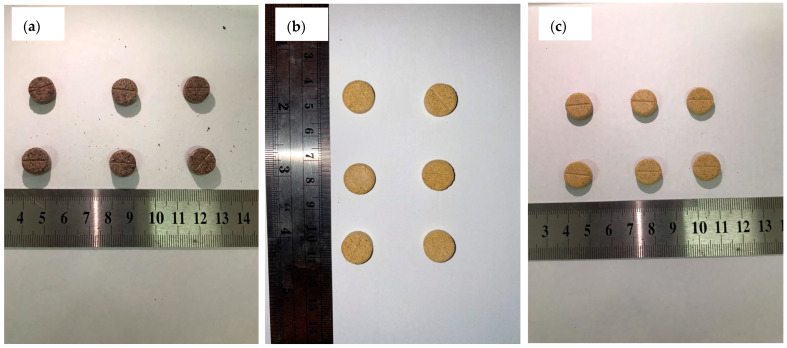
Tablet appearance of formula F1 (**a**), formula F2 (**b**), and formula F3 (**c**).

The three test formulas produced uniform tablet colors. However, in formula F1, the tablet was dark brown and less attractive (see Figure 4a), while formulas F2 and F3 produced a yellowish-orange color (see Figure 4b or Figure 4c). Based on its shape, the F3 formula produced a rounded shape that was neater and firmer than the other formulas. This may be due to the low hardness and friability of the tablets in formulas F2 and F3 (see Table 5).

The hardness test was carried out to determine whether the tablet can survive and avoid damage during packaging and distribution [36]. Tablet hardness should be in the range of 4–8 kg or 39.2–78.4 N (where 1 kg = 9.8 N) [45]. The results of the hardness and friability test show that the MPEM tablets in the F3 formula have good mechanical integrity [37]. A friability value below 1% indicates that the tablet can withstand abrasion during packaging, transportation, and handling [38]. Tablets must not only be hard and not brittle, but they must also be easily dissolved to provide an immediate effect. This can be proven through the disintegration test. For immediate release tablets, the disintegration time must be under 15 min [46]. The properties of the formulas can be seen in Table 7.

Based on the evaluation results, the formula that produced the best tablet characteristics was the F3 formula. For this reason, the evaluation of the stability and effectiveness of antioxidants was only carried out on the F3 formula.

### 3.5. Stability and Antioxidant Activity of Mangosteen Peel Extract during Preparation and Storage

The stability of α-mangosteen in the fabrication process can be observed through the TLC profile. The retention factor value of α-mangosteen was 0.75, with a blue coloration under the UV 254 lamp and green color under the UV 366 lamp. Based on the TLC profile, it can be seen that MPE, MPEM, and their tablets still contain α-mangosteen, which was indicated by the presence of the same spot at a retention factor value of 0.75 (see Table 8). This indicates that the method employed to transform mangosteen peel powder into tablets does not affect the stability of α-mangosteen.

Antioxidant stability in each intermediate product was also maintained (see Table 7). This was confirmed by statistical analysis using the paired *t*-test method, where the IC50 value for each intermediate product did not change significantly (*p* > 0.05). This shows that the microencapsulation method can maintain antioxidant stability from thermal and mechanical exposure in the tableting process. The IC50 of the products can be seen in Table 9.

MPEM tablets showed good stability with maintained α-mangosteen levels for a month of storage at zone IVB conditions (temperature of 40 °C and RH of 75%). The levels of α-mangosteen on days 0, 15, and 30 were 15.87%, 15.75%, and 15.66%, respectively.

## 4. Conclusions

The microencapsulation method using HMPC as a core mixture and PVA as a coating can maintain stability and increase the solubility of mangosteen peel extract in water. This is demonstrated by the α-mangosteen content and IC50 values that were maintained during tablet production and one month of storage at zone IVB conditions. The obtained microcapsule was 411.2 µm in size and has a shape that tends to be round with a rough surface texture. The flow properties and compressibility of MPEM were fair and significantly improved through granulation. The best granule and tablet formula was a formula containing 5% *w*/*w* PVP K30 as a binder, with the characteristics of the tablet being yellowish-orange, hardness of 87.8 ± 1.398 N, friability of 0.94%, and disintegration time of 25.75 ± 0.676 min. According to the findings, the microencapsulation is an effective technique for improving the solubility and antioxidant stability of mangosteen peel extract during manufacture and storage.

## Figures and Tables

**Table 1:** The optimized tablet formulas.

Composition	Amount (%)			Function
	F1	F2	F3	
Amprotab	5	5	5	Disintegrant
NaCMC	5	-	-	Binder
Starch	-	5	-	Binder
PVP K30	-	-	5	Binder
Mg stearate	2	2	2	Lubricants
Talcum	2	2	2	Glidant
Lactose	Ad 100	Ad 100	Ad 100	Filler

**Table 2:** Phytochemical contents of mangosteen peel extract.

Compound	Mangosteen Peel	
	Simplicia	Extract
Alkaloids	+	+
Flavonoids	+	+
Tannins	+	+
Polyphenol	+	+
Saponins	+	+
Quinone	+	+
Monoterpenes-Sequiterpenes	+	−
Triterpenoids-Steroids	+	−

Description: (+) detected, (−) not detected.

**Table 3:** Standard parameters of mangosteen peel extract.

Parameters		Result
	Sample	Reference [35]
Moisture content (% *w*/*v*)	7.83	<10.8
Total ash content (%)	0.244	<4.4
Acid insoluble ash content (%)	0.069	<0.2
Drying shrinkage (%)	8.49	<10
Specific gravity (g/cm^3^)	0.81	<1

**Table 4:** Total phenolic and α-mangosteen content of mangosteen peel extract, mangosteen peel extract microcapsules, and their tablets.

No.	Parameter	Value
1.	Total phenolic content of mangosteen peel extract	39.87% ± 1.840%
2.	Total phenolic content of MPEM tablets	34.73% ± 0.617%
3.	α-mangosteen content of MPEM tablets	15.68% ± 0.332%
4.	Amount of α-mangosteen in MPEM tablets	~70 mg

Note: the significance of changes in phenolic levels in samples before and after tableting was analyzed using the paired *t*-test (*p* > 0.05).

**Table 5:** Physical properties of mangosteen peel extract microcapsules.

No.	Parameter	Value
1.	Flow properties	32.46 ± 1.73
2.	Angle of repose	43.45 ± 1.79°
3.	Compressibility	21.51 ± 0.59
4.	Loss on drying	0.74% ± 0.11%

**Table 6:** Granule properties of the optimized formulas.

No	Parameters	Formula		
		F1	F2	F3
1.	Loss on drying (%)	3.64 ± 0.35	2.14 ± 0.72	1.74 ± 0.05
2.	Flowability (g/s)	3.79 ± 0.29	7.04 ± 0.58	10.74 ± 0.56
3.	Angle of repose (°)	24.21 ± 1.72	25.06 ± 4.17	25.06 ± 0.72
4.	Carr’s index	18.00 ± 3.60	17.00 ± 2.08	9.00 ± 1.52

**Table 7:** Tablet properties of the optimized formulas.

No	Parameters	Formula		
		F1	F2	F3
1.	Organoleptic	Dark brown, still bitter, typical mangosteen aroma, and non-uniform shape	Yellowish orange, less bitter, typical mangosteen aroma, and more uniform shape	Yellowish orange, less bitter, typical mangosteen aroma, and uniform shape
2.	Hardness test (N)	22.45 ± 3.38	36.40 ± 7.18	87.80 ± 1.39
3.	Weight (mg)	0.81 ± 0.00	0.71 ± 0.00	0.77 ± 0.01
4.	Diameter (cm)	0.90 ± 0.00	0.90 ± 0.00	0.90 ± 0.00
5.	Thickness (cm)	0.34 ± 0.01	0.34 ± 0.04	0.33 ± 0.00
6.	Friability test (%)	60	26.39	0.94
7.	Disintegration test (min)	43.04 ± 1.72	10.86 ± 0.45	14.29 ± 0.67

**Table 8:** Thin-layer chromatography profile of each fabrication raw product.

Rf Value	Spots’ Color							
	UV 254				UV 366			
	α-M	MPE	MPEM	Tablet	α-M	MPE	MPEM	Tablet
0.2	-	Blue	-	-	-	Green	Green	-
0.25	-	Blue	-	-	-	Green	-	-
0.3	-	-	Black	Black	-	-	Green	Green
0.5	-	-	-	-	-	Green	-	-
0.75	Blue	Blue	Blue	Blue	Green	Green	Green	Green
0.87	-	-	-	-	-	Green	-	-
0.97	-	-	-	-	-	Green	-	-

Note: α-M = α-mangosteen standard, MPE = mangosteen peel extract, MPEM = mangosteen peel extract microcapsule.

**Table 9:** The IC50 value of each intermediate product compared to ascorbic acid.

Sample	IC50 (µg/mL)
Ascorbic acid	1.81 ± 0.09
Mangosteen peel extract	34.64 ± 6.58
Mangosteen peel extract microcapsule	40.68 ± 0.17
Tablet	41.16 ± 0.69

Note: the significance of changes in IC50 values of samples was analyzed using the paired *t*-test (*p* > 0.05).

## Data Availability

Not applicable.
